# Supplementary material for: Are carbon emissions trading and green financial instruments synergistic? -Comprehensive quantitative research based on content analysis
Source: PLoS One. 2024 Mar 7;19(3):e0298601. doi: 10.1371/journal.pone.0298601 (PMC10919601; doi:10.1371/journal.pone.0298601)
Supplement: S1 Data — (ZIP) [file pone.0298601.s001.zip › Data/地级市Z_F工作报告.zip!/╜¡╦╒/╠⌐╓▌╩╨/└┤╫╘╣1⁄2╓┌║┼í╛┬φ┐╦╩2╛▌═°í┐_2007.docx]

各位代表:

现在，我代表市人民政府向大会作工作报告，请予审议，并请市政协委员和其他列席人员提出意见。

一、2006年工作回顾

过去的一年，我们在中共泰州市委领导下，认真贯彻落实科学发展观和构建和谐社会的重大战略思想，扎实推进全市经济、政治、文化与社会建设，各项工作取得较好成绩，圆满完成了市二届人大四次会议确定的目标任务，顺利实现了“十一五”发展的良好开局。

综合实力得到新提升。全年预计实现地区生产总值突破千亿元大关，达1002.5亿元，增长15.1%，增幅为建市以来最高水平。其中，第一产业增加值95.1亿元，增长4.6%；第二产业增加值585.6亿元，增长16.9%；服务业增加值321.8亿元，增长15.5%。继续推进农业结构调整，优质水稻、弱筋小麦、特种水产等优势农产品基地建设得到加强，特色高效农业生产面积扩大到85万亩，夏粮单产再创历史新高。健全和完善良种繁育推广体系、农业病虫害防控体系、农产品标准化体系，农业科技服务成效明显。四个辖市全部通过省级生态农业县（市）验收。支持和促进农业产业化龙头企业加快发展，龙头企业对基地和农民的带动作用进一步增强。加大农业对外招商力度，利用“三资”达到33.6亿元，增长52.7%。认真实施“第一方略”，促进工业提速增效。规模以上工业完成增加值441.3亿元，增长21.6%，增幅列全省第4位；实现销售收入1589.6亿元、利税139.4亿元，分别增长35%和31.3%。适时引导优势产业、重点企业积极应对宏观环境变化，医药、机电、化工、船舶等四大产业和30强重点企业发展势头良好。全市新增销售过亿元的企业71家，其中过10亿元的6家。支持和服务企业扩大有效投入，全年工业技改财务发生数达306.6亿元，增长47.4%；实施投入亿元以上重大技改项目60个。积极推进名牌战略，新增中国名牌产品2个、国家免检产品12个。泰兴市被表彰为“国家质量兴市先进市”。建筑业继续保持较快发展，完成总产值502亿元，增长19.9%。江苏一建等4家企业进入全省建筑业综合实力20强。建立和落实市及市（区）服务业发展引导资金，突出抓好30个服务业重点项目建设，服务业增加值占GDP的比重较上年提高1个百分点。区域物流、城市商贸、社区服务、旅游等行业呈现出较强发展势头，服务业引进外资有了新的突破。全年完成社会消费品零售总额271.3亿元，增长16%。财税金融工作取得新成绩。全市完成财政收入155.4亿元，增长36.4%，其中地方一般预算收入61.5亿元，增长31.1%，增幅列全省第4位。全年新增贷款首次突破百亿元，达106.5亿元，增幅高出全省平均水平2.1个百分点。南京商业银行泰州分行成立。

富民安民取得新进展。牢固确立“发展经济、造福百姓”的理念，努力让人民群众从改革发展中得到更多实惠。大力推动全民创业，民营经济蓬勃发展。全年新增私营企业4584家、个体工商户3.42万户，净增注册资本138.3亿元；到年底，注册资本3000万元以上的私营企业达308家，其中亿元以上的51家。民营经济实现增加值占GDP的比重首次超过50%。出台新一轮促进就业政策，开展“充分就业社区”创建活动，千方百计扩大社会就业。全年城镇新增就业人员4.77万人，失业人员再就业2.72万人，新转移农村劳动力7.83万人，均超额完成年度目标任务。城镇登记失业率为3.3％。继续完善“五保合一”运作机制，强化社会保险扩面征缴，新增各类参保人员10万人。出台市区居民医疗保险试行办法和计划生育社会保障办法，完善社会保障体系。调高城镇职工最低工资标准和城镇居民最低生活保障标准，建成全省首家企业退休人员老年公寓。启动廉租住房保障工作，开工建设经济适用房12万平方米。推进农民健康工程，农村新型合作医疗覆盖率达93.2%。实施农民工平安计划，推动工伤保险在乡镇用人单位的全覆盖。认真做好被征地农民社会保障和进城务工农民维权工作。高度重视社会困难群体的生活安排，城市低保实现应保尽保，农村低保实行社会化发放；精心组织结对帮扶活动，救助市区特困家庭1026户，扶持农村贫困户8400户。坚持每年为城乡居民办实事，建立健全工作推进机制，为市区人民办的10件实事基本完成，农村新“5+1”实事工程完成年度目标任务。全年城镇居民人均可支配收入12682元，农民人均纯收入5650元，分别增长14％和10.7％；年末，城乡居民储蓄存款余额比年初增加58.8亿元。

改革开放实现新突破。围绕增强经济发展的动力和活力，坚持不懈地深化改革、扩大开放。全市经营性服务类事业单位改制任务全面完成，乡镇建筑企业改制基本到位，外贸、交通企业改革步伐加快，公益类事业单位改革试点已经展开。兴达国际控股有限公司在香港成功上市。推进农村信用社体制改革，姜堰市、兴化市农村合作银行成立。企业投资项目实行核准制和备案制，企业投资自主权进一步得到落实。积极稳妥地开展乡镇综合配套改革试点，为全面推开积累了经验。注重发挥沿江优势、产业优势和企业优势，着力招引产业关联度强、科技含量高、投资规模大的外资项目。全市注册协议外资12.1亿美元，增长66.4%；预计实际利用外资7亿美元以上，增长50%以上。沿江地区逐步成为对外开放的主阵地，在建投资过亿美元或10亿元以上的重大产业项目9个，12个投资超亿元产业项目建成投产；泰州电厂一期工程进展顺利，泰州长江公路大桥项目建议书通过国家审批，沿江高等级公路泰兴段、高港段和靖江段一期工程贯通，泰州港永安港区万吨级公用码头建成运营。医药高新技术产业园建设进展顺利，江阴—靖江工业园区进入省级经济开发区行列。省级开发园区集聚资本的能力进一步提升，注册协议外资、实际利用外资占全市的比重分别达67.2%和74.8%。全市外贸、外经保持快速增长，完成自营出口额22亿美元，增长64.3%，增幅列全省第2位；新签对外承包劳务合同额2.2亿美元，完成营业额2.4亿美元，分别增长31.4%和44.1%。医药出口基地顺利通过审定，成为全国首批、江苏唯一入选的“国家科技兴贸出口创新基地”。靖江市被列为“江苏省船舶出口基地”。泰州边检站开检运行，口岸远程监控系统建成，“大通关”建设取得新进展。内、外交流与合作迈上新台阶，成功承办亚洲博鳌论坛国际医药产业大会和“长三角”城市经济协调会第七次会议，向国内外展示了新泰州，提升了知名度。

城乡面貌发生新变化。注重统筹城乡建设，努力实现协调发展。扎实推进城乡规划全覆盖，城市近期建设用地控规覆盖率达90%以上。以“十大重点工程”为抓手，加快新区建设和老城改造，中心城市建设投资达75亿元。“东大门”改造基本完成，青年路改造一期工程竣工、二期工程提前实施，乔园改造一期、坡子街商业中心改造主体工程基本竣工，体育中心二期、凤城河二期工程开工建设，市文化中心、新区道路南延工程实施方案编制完成，稻河路西侧街区、招贤新村和福寿苑二期改造工程全面启动，新老城区污水管网截流工程完成年度建设任务，扬子江路二期、高港生态公园建成。继续推进“五城同创”，城市管理水平进一步提升。创建省级园林城市获得成功，创建国家环保模范城市通过国家考核验收，创建国家卫生城市通过国家技术考核。强力推进拆除违法建设专项行动，建设环境整治工作取得初步成效。各辖市以城市创建为抓手，坚持加大投入与强化管理并重、加快开发与营造环境并举，城区功能和形象有了较快提升。启动实施“十强争先、百村示范、千村小康”工程，有序推进新农村建设。强化村庄规划编制工作，示范村村庄建设取得积极进展。沈高镇河横村成为全省唯一的“全国部省共建新农村建设示范点”，俞垛镇何野村、华港镇李家庄村成为“全国新农村建设百村联系点”。加快城乡重大基础设施建设，兴泰一级公路开工建设，姜高路改造、姜八公路泰兴段和邮兴盐公路兴化段扩建按期完成，农村公路新建、改造1235公里；区域供水方案确定，泰东河二期工程全面完工，海陵城区西北片防洪封闭工程和南城河、中子河等河道整治工程竣工；兴化唐子、姜堰王石等5项重点输变电工程建成投运，信息化基础设施建设进一步加快，苏中无线电监测中心建成。全年，全社会固定资产投资完成532.6亿元，增长32.1%。

社会发展迈出新步伐。在推动经济加快发展的同时，注重发展各项社会事业。继续实施“科教兴市”战略，积极打造创新型城市。“百企三创”工程全面实施，春兰集团等两家企业被列为“国家级企业技术中心”，扬子江药业集团被列为“全国首批创新型试点企业”。市高新技术创业服务中心被评为“全国先进科技产业园”，市科协被表彰为“全国科普活动先进单位”，兴化市被列为“全国科技富民强县专项行动计划试点市”。全年组织实施省以上火炬、星火计划项目72项，新认定省级高新技术企业34家、高新技术产品71个。加强人才高地建设，引进各类人才12876人。高等教育事业加快发展，高教园区一期工程建成，南师大泰州学院、南理工泰州科技学院实现独立办学。高中教育教学质量不断提高，高考万人本科进线率列全省第4位。义务教育阶段免缴学杂费的政策得到全面落实。职业教育工作继续加强，泰州高职院通过教育部组织的高职高专人才培养工作评估。溱潼会船、靖江讲经等5个项目入选首批省级非物质文化遗产名录。海军诞生地旧址、城隍庙、天目山遗址被列为国家级文物保护单位，实现了我市国家级文物保护单位零的突破。黄桥镇、溱潼镇被命名为“全国历史文化名镇”。加强公共卫生体系建设，市急救中心正式运行，市传染病医院一期工程建成，市卫生监督所大楼投入使用。泰州市惠民医院成立，“快乐从心开始”、“为了母亲的微笑”和“关爱女孩行动”等医疗救助活动产生了良好的社会效应。低生育水平继续保持，计划生育率达98.6％。靖江市、姜堰市、兴化市和高港区被评为“全省‘十五’人口计生工作示范市（区）”。组织实施“万村体育健身工程”，62.8%的行政村建成公共体育设施。参加第十六届省运会并取得优异成绩，综合排名前移两位。大力开展循环经济示范企业、行业和工业园区试点，鼓励和支持全社会开展以节水、节地和节能为重点的节约活动。预计万元GDP能耗下降完成年度目标。以基本农田为核心的耕地资源保护工作得到加强，环保信访听证处理经验在全国推广。史志档案、社会科学、新闻、统计、气象、防震和民族宗教、妇女儿童、残疾人等工作继续加强，质监、药监、工商管理和对台、外事、侨务在服务地方经济发展中取得新成绩。开展纪念地级泰州市成立十周年系列活动、社会主义荣辱观宣传教育活动和城乡文明共建活动，定期举办“百姓议事园”、“百姓大学堂”，为建设和谐美好新泰州营造了良好氛围。组织开展以财务公开、事务公议、政务公示为主要内容的村务公开活动，村民议事和民主评议制度建立健全。贯彻落实《建设法治泰州实施纲要》，打造“法治泰州”。启动“五五”普法工作。法律援助大厅建成。以规范执法行为、促进公正执法为主题的专项整改活动取得积极成效。高港区被表彰为“‘法治江苏合格县（市、区）’创建工作先进单位”。加强国防教育，民兵预备役、双拥工作和人民防空事业取得新成绩。推进“平安泰州”建设，维护社会稳定的预警机制和应对突发事件的处置机制开始建立。预防青少年违法犯罪工作有效开展，社区矫正工作质量稳步提高。泰州市被省批准为“社会治安安全市”，泰兴市、姜堰市、兴化市、海陵区、高港区被省批准为“社会治安安全县（市、区）”。建立、健全安全生产领导责任制，安全生产形势基本平稳。进一步做好信访工作，开展领导干部下访活动，完善“大调解”机制，各类信访案件和矛盾纠纷得到妥善处置，“和谐泰州”建设取得新进展。

自身建设取得新成绩。注重研究和分析宏观经济形势，适时提出政策性措施、指导性意见，着力化解发展中遇到的矛盾和制约。强化目标责任意识，加强实施过程监控，扎实组织推进“411”工程。完善行政决策机制，重大事项做到广泛征求社会各界意见。自觉接受市人大的法律监督、工作监督和市政协的民主监督，重视和发挥各民主党派、工商联、人民团体的参政议政作用。认真落实《关于进一步发挥市人大代表作用加强市人大常委会制度建设的若干意见》，健全联系市人大代表和市政协委员工作制度，负责、高效地办理人大代表建议和政协委员提案。继续强化行政效能监察，主动接受社会舆论的监督。贯彻实施《公务员法》，强化人员培训和交流，公务员队伍整体素质不断提高。深入开展“人民满意机关”创建和“为发展服务，让群众得益”的基层站所作风建设活动，创建了一批机关服务品牌。加快电子政务建设，扩大政务公开，市行政服务中心网上审批系统建成开通。党风廉政建设取得新成效。严格执行廉政建设责任制，领导干部廉洁自律工作进一步加强。扎实推进治理商业贿赂专项工作，切实加强政府重点项目审计监督。认真办好市长公开电话、市长信箱和行风热线，及时为市民解决实际困难和问题。加强机关事务管理，保障能力得到增强。

各位代表，过去一年政府各项工作取得的成绩，是在中共泰州市委的正确领导下，在市人大、市政协的监督和支持下，全市人民团结拼搏、共同努力的结果。在此，我代表市人民政府，向辛勤工作在全市各行各业的广大干部群众，向给予政府工作热情支持和有力监督的全体人大代表、政协委员，向各民主党派、工商联、无党派爱国人士、各人民团体和广大离退休老同志，向中央和省驻泰机构以及驻泰部队指战员、武警官兵和公安干警，向所有关心支持泰州现代化建设的海内外朋友，表示衷心的感谢！

过去的一年，全市经济社会发展中也存在不少矛盾和问题。一是农民增收的长效机制有待建立和完善；二是经济结构调整的任务仍很艰巨；三是开放型经济、民营经济发展的层次和水平尚需进一步提升；四是中心城市的功能还不够完善；五是社会建设和管理也有待进一步加强，等等。我们一定高度重视这些问题，采取有效措施，逐步加以解决。

二、2007年工作任务

今年是贯彻市第三次党代会精神的起始之年，是实施“十一五”发展规划的关键之年，也是完成本届政府任期目标的攻坚之年。做好今年的政府工作，意义更加重大。

今年政府工作的总体要求是：以邓小平理论、“三个代表”重要思想和胡锦涛总书记的重要指示为指导，以科学发展观统领经济社会发展全局，深入贯彻党的十六届六中全会和市第三次党代会精神，坚持科学发展，转变增长方式，统筹城乡建设，推进开放开发，着力改善民生，构建和谐社会，努力实现经济社会又好又快发展，确保全面完成本届政府任期目标，加快“全面达小康、建设新泰州”进程。

今年全市国民经济和社会发展主要调控目标初步安排为：地区生产总值增长12%以上，其中第一产业、第二产业、服务业增加值分别增长4%、15%和13%；财政收入增长18%，其中地方一般预算收入增长18％；全社会固定资产投资增长20%；社会消费品零售总额增长14%；自营出口额增长20%；实际利用外资8亿美元；城镇居民人均可支配收入增长10%，农民人均纯收入增长9%；居民消费价格涨幅控制在3%左右；城镇登记失业率控制在4%以内；万元GDP能耗下降4.4%。

今年，我们将着重做好五个方面的工作：

1.着力转变经济增长方式，切实提升产业发展层次。转变经济增长方式是泰州科学发展的必然要求，提升产业发展层次是泰州率先发展的迫切需要。必须进一步创新发展理念、发展思路、发展途径，扎实推动经济又好又快发展。

大力发展现代制造业。坚定不移地走新型工业化道路，加快建设区域特色明显、比较优势凸现的现代制造业基地。围绕促进优势产业集聚，注重发挥产业规划、政策及投资目录的导向作用，鼓励支持产业、企业向沿江、沿靖盐路、沿兴泰线产业带加快集结，推动医药、机电、化工、船舶等四大产业基地发展壮大，推动不锈钢制品等十大特色产业集群发展。围绕促进重点企业发展，引导、支持重点企业加快主题园区建设、加大品牌培育力度、加强企业上市工作，进一步提升规模企业发展水平，着手打造全市50强。同时，组织实施中小企业成长工程，重点扶持成长型企业100家，新增规模企业300家以上。围绕促进有效投入扩大，完善服务重大技改项目建设的协调推进机制，推动项目快获批、快开工、快投产。全年投入过亿元的重点技改项目60个以上，工业技改财务发生数增长25%以上。在扎实推进金融生态环境建设的同时，继续加大对重点企业和重点项目的信贷支持力度，认真解决中小企业贷款难的问题。

积极发展现代服务业。把加快服务业发展摆上突出位置，组织实施“833工程”，确保服务业增速快于GDP增速，力争占比提高1个百分点。整合铁公水、路港桥物流资源，引进和培育一批现代物流企业，重点抓好泰州港综合物流园区、海陵城北物流中心、靖江三江物流中心等物流集聚区建设。培育和壮大海陵城区东部市场、泰兴北二环专业市场群等重点市场、特色市场，大力促进医药高新技术产业园会展交易区、华东五金城、戴南不锈钢交易城等市场集聚区发展，积极新建一批大型产品交易市场。进一步繁荣城市商贸业，加快坡子街商业中心、兴化宝都建材城等重点项目建设，推动社区连锁、便利店发展，加快农副产品市场改造。组织实施《泰州市旅游业发展总体规划》，推进溱湖风景区等重点景区建设，鼓励发展乡村游等特色项目，加快把旅游业培育成为新兴支柱产业。继续促进保险、信息等产业发展，规范各类中介服务机构。

努力打造创新型城市。把增强自主创新能力作为泰州发展的主要战略支撑，加快实现投资拉动向创新驱动、资源依赖向科技依托、泰州制造向泰州创造的转变。组织实施“百企三创”、“科技富民”和“专利推进”三大工程，推动全社会科技创新实践。鼓励、支持企业工程技术研究中心等科研开发机构建设，加快市及辖市（区）创业服务中心发展，启动中国科学院泰州应用技术研发及产业化中心、软件园和江苏（泰州）新药研究院、清洁能源研究院建设，打造更多的科技创新平台。推进人才高地建设，重点培养、引进一批善于组织研发和成果转化的科技企业家、学科带头人和留学归国人员，提升区域科技创新能力。贯彻实施《全民科学素质行动计划纲要》，继续抓好科学普及工作。

加快建设资源节约型、环境友好型社会。认真贯彻落实节约优先、环保优先的方针，努力实现节约发展、清洁发展、安全发展和可持续发展。加强对重点行业的能耗监控，突出抓好耗能大户的节能工作，从严控制新上高耗能项目。强化对项目用地的审核，促进土地合理、集约和可持续利用。注重环境容量问题，严格执行环境准入门槛和环保标准，从源头上控制污染物排放。抓好环境综合治理，开展整治违法排污企业、化工生产企业和集中式饮用水源专项行动。大力发展循环经济，鼓励企业循环式生产，促进资源循环利用。开展全国土壤污染状况调查和全国第一次污染源普查工作。实施《生态市建设规划纲要》，在积极创建国家环保模范城市群的基础上，启动生态市创建工作。

2.着力统筹城乡协调发展，扎实推进新农村建设。坚持以发展生产为中心，以农民增收为核心，从农民要求最迫切、受益最直接的事情抓起，加快新农村建设，促进农业增效、农民增收、农村发展。

大力发展现代农业。把发展“高产、优质、高效、生态、安全”的现代农业作为推进新农村建设的主要着力点，不断提高农业综合生产能力。继续优化农业布局、品种结构，重点打造弱筋小麦、加工型蔬菜、特色水产等十大特色产业，建成高效农业面积100万亩、规模畜禽养殖小区20个。推动农业科技服务进村入户，引进推广新品种50个以上。加强农产品品牌建设，促进优势农产品提质升档，力争新认证无公害农产品和绿色、有机食品50个以上。支持农业产业化龙头企业发展，新建各类农民专业合作经济组织100个，发展订单农业基地面积200万亩。强化农产品质量监管，提高本地农产品供给的安全程度，确保无重大动物疫病流行、无重大农产品质量安全事件和大面积农作物病虫害发生。推进“绿色泰州”建设，全市森林覆盖率提高1.2个百分点。

抓好镇村建设和发展。深入开展乡（镇）学戴南张郭、村学华西活动，继续推进新农村建设。组织实施城镇体系规划、镇村布局规划，加快编制村庄建设规划，有效指导重点中心镇、小康村、农民集中居住点建设和发展。引导16个重点中心镇科学修编2020版总体规划，加快打造一批工业经济强镇、商贸流通重镇、文化旅游名镇。继续实施“十强争先、百村示范、千村小康”工程，注重发展农村社会事业，改善农村公共服务，着力研究和解决基层、群众急需解决的突出问题。注重彰显个性，整治、改造、提升原生态的特色村。全年力争建成省级康居示范村10个、市级小康试点示范村100个。在保护耕地、维护农民权益的前提下，引导农村工业企业加快向园区集中、农民居住逐步向新型社区集中、农业土地有序向适度规模经营集中。完善城乡帮促机制，深入推进“221”工程，着力增强经济薄弱乡镇的内生发展动力。

办好农村新“5+1”实事。实施农村道路通达工程，全面完成上一轮通村公路建设任务，启动新一轮农村道路建设。实施农村“三清”工程，推广使用清洁能源，加快农村户厕改造，抓好村庄综合环境整治试点。实施百万农民培训工程，加强农业实用技术培训、农村劳动力转移培训和农民创业培训，新转移农村劳动力5万人以上。实施农民健康工程，推进农村“二次改水”，加强农村公共卫生和基本医疗服务体系建设，提高农村新型合作医疗保障水平。实施农村文化建设工程，加强村、社区文化活动室建设，推进有线电视“组组通”，组织好“三下乡活动”。启动农村社会基本养老保险工作，抓好100个村试点。

3.着力增强经济发展活力，进一步提高开放型经济发展水平。发展开放型经济，是泰州快速崛起的重要引擎。必须坚定不移地扩大对外开放，全力推进开放型经济加速发展。

以沿江开发为重点，力求招商引资新突破。适应开放环境新变化、外资进入新趋势，努力提升沿江开发、招商引资和园区发展水平。以更大力度推进沿江产业招商，支持泰州电厂一期等重大在建项目和中海油、东泰石化等重大开工项目建设，加快沿江公共码头、污水处理、供电供热等配套设施建设。按照务求实效的要求，精心组织好赴日韩、美加招商和对台招商，积极参与“欧盟招商周”、厦洽会等重大招商活动，办好第六届泰州科技经贸洽谈会。贯彻量质并重的方针，以开展“项目双促年”为抓手，在扩大引资总量的同时，注重提升引资质量，积极引进处在产业链高端环节，投向农业、服务业、节能环保等领域以及属于跨国公司研发机构、制造企业、营销中心的外资项目。注重在更高层次上推进开发园区建设和发展，力争在培育园区特色产业、提升产业配套能力、完善基础设施功能等方面取得新突破，促进重点开发园区加快成为先进产业的集聚区、科技创新的先导区。加快医药高新技术产业园发展，确保一期项目基本建成、二期项目开工建设。组织实施“6+6”工程，力争农业综合开发示范区建设取得新进展。

以全面“入世”为契机，促进外贸、外经快速发展。积极应对“入世”过渡期结束带来的机遇和挑战，加快转变外贸增长方式，保持外贸出口快速增长。实施科技兴贸、以质取胜和市场多元化战略，扩大高新技术产品、机电产品和绿色农产品出口。培育出口企业品牌，新增部、省重点支持的出口品牌2至3个。用好“国家科技兴贸出口创新基地”平台和相关政策，积极申报国家出口加工区，促进和扩大进出口贸易。鼓励企业发挥“建筑之乡”优势，积极“走出去”，扩大境外工程承包和劳务输出。重视和做好外事、侨务、对台和口岸工作。

以融入“长三角”为方向，加强区域经济合作与交流。按照“接轨上海、融入苏南、吸纳浙商”的思路，积极参与“长三角”区域经济合作与交流。发挥本地产业、资源、区位等比较优势，更加主动地争取上海的产业转移、服务辐射和科技成果转化。围绕构建锡常泰城市群，加强与苏南的基础设施连接、产业分工对接和城市功能衔接，促进交通联网、旅游联手、信息联通、生态联保；放大靖江与江阴联动开发效应，支持沿江地区与苏南联动发展。继续强化招引浙商工作，注重吸纳规模实力型浙商、技术资本复合型浙商。

4.着力推进城市现代化，努力塑造中心城市新形象。城市是现代经济社会发展的主要载体和带动力量。必须始终立足市情实际，坚持不懈地提升中心城市的首位度和辐射力。

加快中心城市建设。坚持“南进、东扩”的发展方向，以“十大重点工程”为抓手，继续加大中心城市建设力度。加快建设新城区，开工建设新区道路南延、文化中心工程，实施高教园区二期工程，启动周山河街区一期建设，进一步拉开框架、扩大规模。继续改造老城区，加快城北地区改造步伐，启动城北污水处理厂及配套管网一期工程，推进凤城河风景区二期工程，完成梅兰芳纪念馆一期改造，继续实施城区道路、街区改造和河道整治，加快“城中村”改造和“城中厂”搬迁。积极发展港区，建设金港南路商业街区，完成台湾商城二期工程，实施雕花楼二期工程。启动绿博园规划和建设。注重引导各辖市加快城区建设，不断完善功能、提升形象。

提高城市管理水平。坚持规划先行，引入先进的规划理念，推进城市近期建设用地控规全覆盖。筹建城市规划展示馆，搭建市民参与城市规划的新平台。完善城市管理长效机制，推进管理重心下移和事权下放，提高城市管理综合执法效能。继续抓好建设环境、交通环境和市容环境的综合整治。遵循市场化运作规律，积极创新市容卫生、园林绿化、公共交通的管理体制和运作机制。巩固扩大“五城同创”成果，启动国家园林城市创建工作，力争创建成国家卫生城市和中国优秀旅游城市。

加强重大基础设施建设。按照适度超前、配套完善的要求，加快基础设施网络化、现代化进程。进一步完善交通网络，开工建设泰州长江公路大桥和江海高速泰州段、大兴金公路兴化段，实施兴泰一级公路建设，贯通沿江高等级公路，新建、改造农村公路1000公里。启动南水北调水源调整、淮河流域洼地治理及卤汀河拓浚、盐靖河整治工程，继续推进海陵城区城市防洪工程建设和水环境综合整治。实施区域供水工程，加快解决部分辖市居民饮用长江水问题。启动同济、沈星输变电工程建设，完成洋思变扩建工程；实施10千伏及以下电网新建改造，支持新农村建设。移建泰州中波台，加快固定电话、移动电话网络扩容工程和增值业务平台建设。

5.着力构建和谐社会，不断加强社会建设和管理。认真贯彻落实“统筹发展、兼顾利益、重在建设”的方针，高度重视社会建设，切实加强社会管理，大力促进社会发展，努力建设和谐社会。

进一步提高人民生活水平。坚持以人为本，着力改善民生。把支持创业作为致富百姓的首要途径，强化创业培训指导、贷款担保和政策支持，推动民众自主创业，大力发展民营经济。全年民营经济新增注册资本100亿元以上。把实现相对充分就业作为重要的社会发展目标，认真抓好全国城乡统筹就业试点工作，健全完善就业再就业援助制度，促进下岗失业人员稳定就业、大龄就业困难群体和被征地无业农民再就业、残疾人按比例安排就业。全年新增就业岗位5万个。把建立和完善覆盖城乡居民的社会保障体系作为一项重要任务，落实灵活就业人员和新办民营企业参加社会保险补贴政策，创新社会助保和断保补助机制；建立退休人员参加医疗保险多渠道筹资机制，抓好城镇医疗保险和农村合作医疗“政策接轨”试点。把解决好社会困难群体的实际问题摆上工作的突出位置，继续做好城乡居民最低生活保障和被征地农民社会保障工作，完善大病救助和突发安全事件受伤人员救助机制，进一步扩大廉租房覆盖面，扎实抓好扶贫济困工作，积极发展社会福利和各项慈善事业。把完善工资协商和调解机制作为企业深化改革的重要内容，切实维护劳动者的合法权益。

大力发展各项社会事业。适应构建和谐社会新要求，进一步加大财政对基础教育、公共卫生、公益文化、群众体育的投入力度，促进城乡、区域社会事业协调发展。推进义务教育均衡发展，加快义务教育学校“四配套工程”建设。继续调整普通高中布局，确保65%以上的学生进入优质高中接受教育，高中教育阶段毛入学率80%以上。继续扩大本科院校的招生规模，全市高校在校生力争3万人以上。发展职业教育，整合职教资源，积极培养适用、急需技能人才。重视文化事业和文化产业发展，保障人民群众普遍享有基本公共文化服务。完成学政试院布馆工作。加强城乡社区卫生服务机构建设，进一步提高医疗卫生的服务质量和水平。强化食品、药品监督，保证群众饮食、用药安全。加快体育中心二期工程建设，承办好中国式摔跤国际邀请赛。继续稳定低生育水平，重视出生人口性别比例偏高和人口老龄化问题。完成第二次全国农业普查年度工作。积极发展史志档案、社会科学、新闻、统计、气象、防震等事业，认真做好民族宗教、妇女儿童、残疾人等工作。

加强精神文明和民主法制建设。以创建全国文明城市工作先进市为抓手，扎实推进城市文明创建工作。认真贯彻落实《公民道德建设实施纲要》，深入开展社会主义荣辱观宣传和实践活动，着力提升全民素质。以社区、村镇为重点，推进城乡文明共建活动，培育健康文明的社会风尚。切实做好国防教育、民兵预备役和兵员征集工作，争创全国双拥模范城“二连冠”。继续推进村务公开，进一步充实公开内容、完善公开程序、创新公开形式、健全监督机制，扩大基层民主。开展村民自治模范单位创建活动，启动第八届村委会换届选举工作。加强城市社区建设，完善社区自治功能。认真组织开展“法治江苏合格县（市、区）”创建活动，推进“法治泰州”建设。扎实抓好以宪法为核心的“五五”普法，积极开展法律服务和法律援助。以创建“全国科技强警示范市”为抓手，开展新一轮“平安泰州”建设。健全人民调解与司法调解、行政调解相结合的“大调解”机制，构建城乡一体的“大防控”体系。建立健全市及辖市两级应急体系，切实提高应急处置能力。强化安全生产工作，遏制重、特大事故发生，保障人民群众生命财产安全。

三、努力建设人民满意政府

各位代表，全面贯彻落实科学发展观，着力构建社会主义和谐社会，对政府工作提出了更高的要求。我们将以打造服务政府、高效政府、法治政府、廉洁政府为主线，进一步转变职能、深化改革、加强管理，为全面完成本届政府任期目标提供有力保障。

1.始终牢记宗旨，自觉为民勤政。政府的权力源于人民，必须做到用权为民、施政为民、勤政为民，对群众负责，受群众监督，让群众满意。始终坚持把维护、实现、发展好广大人民群众的根本利益作为政府工作的出发点和落脚点，在发展经济中更加注重富裕群众，在城市建设中更加注重方便群众，在利益调整中更加注重维护群众。自觉从群众最期盼的事情做起，始终带着感情和责任，经常深入基层和群众，着力为市民办好扶贫帮困、社会保障、经济适用房建设等10件实事；继续办好市长公开电话、市长信箱和行风热线，进一步畅通群众反映诉求的通道。把坚持领导干部信访接待和下访工作制度作为自身建设的重要内容，深入贯彻信访条例，认真落实领导责任制，及时、有效地处置和化解各种社会矛盾，维护社会政治稳定。坚持以建设服务型政府、服务型机关为抓手，全面落实首问责任、服务承诺、责任追究等制度，大力推进机关岗位标准化建设，更好地服务基层、企业和群众，使勤政为民成为政府全体工作人员的行为规范和自觉行动。

2.坚持与时俱进，推进科学施政。坚持把科学发展观和构建和谐社会的重大战略思想贯穿于政府工作的全过程和政府管理的各环节，不断提高施政水平。进一步创新行政理念，在继续强化经济调节和市场监管的同时，更加重视加强社会管理、促进社会发展，更加重视提供公共服务、保障公共安全。进一步改进行政管理，继续抓好行政管理体制改革，确保行政审批“两集中、两到位”和“受理、办复一个窗口”；深化财政预算体制改革，着力构建公共财政体制框架；完善决策协商、专家论证和社会公示、听证等制度，建立健全了解民情、反应民意、集中民智的政府决策机制。进一步提高行政效率，加强政府执行力建设，健全抓落实的工作机制，强化效能建设和效能问责；加快电子政务建设，完善网上办公、咨询和审批系统，推进“无纸化”办公。进一步增强行政能力，大力开展学习型机关、学习型班子和学习型干部争创活动，不断提升政府工作的层次和政府工作人员的综合素质。

3.主动接受监督，促进依法行政。自觉依照《监督法》和《建设法治泰州实施纲要》，行使权力、履行职责、接受监督。坚持向人大及其常委会报告工作和向政协通报情况的制度，自觉接受市人大、市政协的监督，主动听取各民主党派、工商联、人民团体的建议和意见，认真办理人大代表建议和政协委员提案。加强对政府规范性文件的必要性、可行性和合法性审查，建立规范性文件定期清理制度。全面推行行政执法责任制和执法过错责任追究制，强化对行政执法行为的监督，严肃查处违法使用权力侵犯群众利益的行为，做到有权必有责、用权受监督、侵权须赔偿。积极推进“阳光政府”建设，建立政府新闻发布制度，做好全省政务公开试点工作，进一步扩大政务公开和公用事业单位办事公开，保障公众对公共事务的知情权和监督权，增强政府的透明度和公信力。

4.倡导廉洁自律，坚持从严治政。反腐倡廉事关民心向背。坚持教育、制度、监督并重，规范政府工作人员的从政行为。大力开展廉政文化建设活动，引导公务员强化廉洁从政意识，筑牢拒腐防变的思想道德防线。严格执行部门预算、政府采购、建设项目公开招投标和“收支两条线”等各项制度，加大对重点领域、重大工程、重要环节的审计和监察力度，努力从源头上、制度上预防和制止腐败。全面落实廉政建设责任制，严肃查处各种违法违纪案件，推动党风廉政建设继续走在全省前列。切实加强行风建设和专项治理，着力解决群众反映强烈的突出问题，树立清正廉洁的良好政风。

各位代表，时代赋予我们重任，人民寄予我们厚望。让我们紧密团结在以胡锦涛同志为总书记的党中央周围，认真贯彻十六届六中全会和市第三次党代会精神，全面贯彻落实科学发展观，在中共泰州市委的领导下，紧紧依靠全市人民，与时俱进，开拓创新，励精图治，扎实工作，确保全面完成和超额完成本届政府任期目标，为“全面达小康、建设新泰州”而团结奋斗！
